# Likelihood of Null Effects of Large NHLBI Clinical Trials Has Increased over Time

**DOI:** 10.1371/journal.pone.0132382

**Published:** 2015-08-05

**Authors:** Robert M. Kaplan, Veronica L. Irvin

**Affiliations:** 1 Agency for Healthcare Research and Quality, U.S. Department of Health and Human Services, Rockville, Maryland, United States of America; 2 Oregon State University, Corvallis, Oregon, United States of America; Mario Negri Institute for Pharmacology Research, ITALY

## Abstract

**Background:**

We explore whether the number of null results in large National Heart Lung, and Blood Institute (NHLBI) funded trials has increased over time.

**Methods:**

We identified all large NHLBI supported RCTs between 1970 and 2012 evaluating drugs or dietary supplements for the treatment or prevention of cardiovascular disease. Trials were included if direct costs >$500,000/year, participants were adult humans, and the primary outcome was cardiovascular risk, disease or death. The 55 trials meeting these criteria were coded for whether they were published prior to or after the year 2000, whether they registered in clinicaltrials.gov prior to publication, used active or placebo comparator, and whether or not the trial had industry co-sponsorship. We tabulated whether the study reported a positive, negative, or null result on the primary outcome variable and for total mortality.

**Results:**

17 of 30 studies (57%) published prior to 2000 showed a significant benefit of intervention on the primary outcome in comparison to only 2 among the 25 (8%) trials published after 2000 (χ^2^=12.2,df= 1, p=0.0005). There has been no change in the proportion of trials that compared treatment to placebo versus active comparator. Industry co-sponsorship was unrelated to the probability of reporting a significant benefit. Pre-registration in clinical trials.gov was strongly associated with the trend toward null findings.

**Conclusions:**

The number NHLBI trials reporting positive results declined after the year 2000. Prospective declaration of outcomes in RCTs, and the adoption of transparent reporting standards, as required by clinicaltrials.gov, may have contributed to the trend toward null findings.

## Introduction

Large randomized clinical trials (RCTs) provide the best evidence to justify new treatments or to identify treatments that do not improve patient outcomes. Gordon and colleagues reported that most large NHLBI-funded trials produce null results[1], but their analysis only considered papers published after 2000. Considering all large trials over the last 40 years, we explore whether there has been a trend toward null finding in recent years and consider potential explanations for trends in observing null outcomes.

## Method

### Sample of Studies

We identified all large RCTs that involved drugs or supplements funded between 1970–2012. To avoid non-publication bias, we focused on large trials where non-reporting of outcomes is rare[1]. The search process is summarized in a PRISMA diagram (S1 Fig). Two independent searches were conducted to improve probability of accurately capturing all related trials–one by the study authors and the second by NHLBI. We searched three different NIH grant databases (QVR, NIH REPORTER, and CRISP) for RCTs that were primarily funded or administered by NHLBI. QVR is an internal NIH data-base, but readers can replicate our search using NIH REPORTER and CRISP which are publically available resources listing all grants and associated publications. Inclusion criteria were: RCT for studies funded from 1970–2012; grants or contracts; direct costs funded were large enough to require special authorization (>$500,000/ year); the word “trial” had to appear in the study objectives or abstract; and primary outcome was cardiovascular risk factor, event or death. Exclusion criteria included: project still active; no human subjects protocol required; pediatric studies; animal studies; non-RCTs (i.e. observational, cohort, case control, genetic or proteomics, measurement, basic clinical research); or interventions that did not involve a drug or supplement (i.e. behavior change, devices, surgeries). An expanded methods section is available in the Supplemental Materials.

We coded the following variables: start year (earliest funding noted), publication year of main outcome study, funded through contract or cooperative agreement from NHLBI, type of comparator (placebo, active comparator, usual care), primary outcome specified or not, CONSORT diagram included in publication, whether funding was exclusively from NIH versus joint industry/NIH funded (including industry contributed medications), and if they had listed any other significant results that were neither the primary outcomes or the side effects of the drug. In addition, we considered whether studies were registered in clinicaltrials.gov prior to publication.

Each trial was categorized as showing significant benefit, null, or significant harm for the primary outcome and for total mortality (See Tables 1 and 2). Null was defined as a confidence interval for the RR that included 1.0.using a two-tailed test with alpha set at 0.05. The analysis was standardized by re-computing the relative risk (RR) with 95% confidence intervals (CI) for all trials.

**Table 1:** Study characteristics and overall effect for main outcome and total mortality for studies not registered in ClinicalTrials.gov prior to publication.

Study	Acronym	Start Year	Pub Year	Primary Outcome (PO)	Primary Outcome	Total Mortality
**Asymptomatic Carotid Artery Progression Study**	ACAPS	1988	1994	3-year change in IMT wall of CA	Benefit	Benefit
**Aspiring Myocardial Infarction Study**	AMIS	1974	1980	All-cause mortality	Null	Null
**Boston Area Anticoagulation Trial for Atrial Fibrillation**	BAATAF	1985	1990	Prevalence of stroke	Benefit	Benefit
**Beta-Blocker Heart Attack Trial**	BHAT	1977	1982	All-cause mortality	Benefit	Benefit
**Cardiac Arrest in Seattle: Conventional Versus Amiodarone Drug Evaluation**	Carotid	1986	1992	>50% restenosis at 1 year	Null	NP
**Cardiac Arrest in Seattle: Conventional Versus Amiodarone Drug Evaluation**	CASCADE	1987	1993	Composite of survival free of various cardiac events	Benefit	Null
**Cardiac Arrhythmia Suppression Trial**	CAST	1986	1991	Cardiac death or arrest	Harm	Harm
**Coronary Drug Project**	CDP	1965	1975	All-cause mortality	Null	Null
**Coronary Intervention Study**	CIS	1971	1984	Progression of CAD	Null	Null
**Cholesterol-lowering atherosclerosis study**	CLAS	1979	1987	Atherosclerosis regression	Benefit	NR
**Coronary Primary Prevention Trial**	CPPT	1971	1984	CHD death or definite non-fatal MI	Null	Null
**Familial Atherosclerosis Treatment Study**	FATS	1984	1990	Regression primary artery	Benefit	NP
**(Fenfluramine & Phentemrine) Cardiovascular System in Obesity: Effect of Treatment**	FEN-PHEN	1983	1992	Weight loss	Benefit	NR
**Fish oil on blood pressure among mild hypertensive subjects**	FISH OIL	1983	1993	Blood pressure	Null	NR
**Hypertension Control Program**	HCP	1980	1987	Normative bp	Benefit	NP
**Hypertension Detection and Follow-up Program**	HDFP	1971	1979	All-cause mortality	Benefit	Benefit
**Hypertension Prevention Trial**	HPT	1981	1990	Composite of on medication or dbp>90 sbp>140	Benefit	NP
**Potassium chloride on blood pressure in hypertensive men**	KCL	1983	1990	Reinstated blood pressure med	Null	NR
**Multicenter Investigation of Limitation of Infarct Size**	MILIS	1977	1984	Infarct size	Null	Null
**Myocardial Infarction Triage and Intervention Trial**	MITIT	1988	1993	Ranked composite of death, stroke, bleeding, infarct size	Null	Null
**Multiple Risk Factor Intervention Trial**	MRFIT	1974	1982	CHD death	Null	Null
**Immunosuppressive therapy for myocarditis**	MYOCARDITIS	1986	1995	Change in left ventricular infarction size	Null	Null
**Postmenopausal Estrogen/Progestin Interventions Study**	PEPI	1994	1995	Lipoproteins, blood pressure	Benefit	NR
**Physician’s Health Study**	Physicians Health Study	1981	1989	MI infarction	Benefit	Null
**Stanford Coronary Risk Intervention Project**	SCRIP	1983	1994	Angiographic change of diameter	Benefit	Null
**Systolic Hypertension in the Elderly Program**	SHEP	1984	1996	Major CVD events	Benefit	Null
**Studies of Left Ventricular Dysfunction**	SOLVD	1975	1991	All-cause mortality	Benefit	Benefit
**Late thrombolytic therapy preserves left ventricular function**	Thrombo	1982	1989	Change in angiographic endpoint ejection fraction	Benefit	Null
**Thrombolysis in myocardial infarction**	TIMI	1983	1985	Reperfusion	Benefit	NP
**Treatment of Mild Hypertension Study**	TOMHS	1985	1993	Major CVD events	Null	NR

**Table 2:** Study characteristics and overall effect for main outcome and total mortality for studies registered in ClinicalTrials.gov prior to publication.

Study	Acronym	Start Year	Pub Year	Primary Outcome (PO)	Primary Outcome	Total Mortality
**Action to Control Cardiovascular Risk in Diabetes—Blood Pressure**	ACCORD-BP	2000	2010	Composite—Non-fatal MI, non-fatal stroke, CVD death	Null	Null
**Action to Control Cardiovascular Risk in Diabetes—Diabetes**	ACCORD-Diabetes	2000	2008	Major or non-fatal MI, non-fatal stroke, CVD death	Null	Harm
**Action to Control Cardiovascular Risk in Diabetes—Lipids**	Accord-Lipid	2000	2010	Major non fatal MI stroke or CVD death	Null	Null
**Azithromycin and Coronary Events Study**	ACES	1998	2005	Composite—death from CV, revacularization, hospitalization	Null	Null
**Atrial Fibrillation Follow-up Investigation of Rhythm Management**	AFFIRM	1995	2002	All-cause mortality	Null	Null
**Atherothrombosis Intervention in Metabolic Syndrome with Low HDL/High Triglycerides Impact on Global Health**	AIM-HIGH	2005	2011	Composite death plus events	Null	Null
**Antihypertensive and Lipid-Lowering Treatment-Amlodipine**	ALLHAT-BP	1993	2002	Fatal or non-fatal MI	Null	Null
**Antihypertensive and Lipid-Lowering Treatment-Doxazosin**	ALLHAT-DOX	1993	2000	Fatal or non-fatal MI	Null	Null
**Antihypertensive and Lipid-Lowering Treatment-Pravastatin**	ALLHAT-LLT	1993	2002	All cause mortality	Null	Null
**Alpha Omega Trial: Study of Omega-3 Fatty Acids and Coronary Mortality**	Alpha Omega	2005	2010	Fatal and non-fatal cardiovascular	Null	Null
**Enhancing Recovery in Coronary Heart Disease Patients**	ENRICHD	1995	2003	Death or recurrent MI	Null	Null
**Estrogen Replacement and Atherosclerosis**	ERA	1994	2000	Mean minimal coronary artery diameter	Null	Null
**Initial Myocardial Metabolic Enhancement During Initial Assessment and Treatment in Emergency care**	IMMEDIATE	2004	2012	Progression of ACS to MI	Null	Null
**Magnesium in Coronaries (MAGIC)**	MAGIC	1998	2002	30 day all cause mortality	Null	Null
**Prevention of Events With Angiotensin-Converting Enzyme Inhibitor Therapy**	PEACE	1995	2004	Death from CVD	Null	Null
**Prevention of Recurrent Venous Thromboembolism**	PREVENT	1998	2003	Recurrent venous thromboembolism	Benefit	Null
**Stop Atherosclerosis in Native Diabetics Study**	SANDS	2002	2008	Carotid artery intimal medial thickness	Benefit	Null
**Sudden Cardiac Death in Heart Failure Trial**	SCD-HeFT	1997	2005	Total mortality	Null	Null
**Women's Antioxidant Cardiovascular**	WACS	1993	2007	CVD death or events	Null	Null
**Women’s Angiographic Vitamin and Estrogen Trial**	WAVE	1996	2002	Change in minimum luminal diameter	Null	Harm
**Women’s Estrogen-Progestin Lipid-Lowering Hormone Atherosclerosis Regression Trial**	WELL-HART	1995	2003	Change in percent stenosis	Null	Null
**Women's Health Initiative- Estrogen**	WHI-E	1999	2004	CHD incidence	Null	Null
**Women's Health Initiative- Estrogen-Progestin**	WHI-EP	1999	2002	CHD incidence	Harm	Null
**Women's Health Study- Aspirin**	WHS-ASA	1991	2005	Non-fatal MI or stroke or death from CVD	Null	Null
**Women's Health Study- Vitamin E**	WHS-E	1991	2005	Events and cardiac interventions.	Null	Null

## Results

Among 4,089 individual years of grant funding, almost half were excluded as multiple years of the same grant and over 20% were excluded because they were single sites in multi-site trials, coordinating centers, or ancillary studies of the same trial. An additional 1,176 grant abstracts did not match our criteria and were excluded (see S1 Table for detailed reasons). Main outcome papers were searched for 84 trials; 10 were not published and 25 did not match search criteria and were excluded (See S1 Fig for the PRISMA diagram and S1 Table for the number of studies excluded by reason.) Following exclusions, we identified a total of 49 funded grants. Four of these grants resulted in multiple unique trials (ACCORD Blood Pressure, Diabetes, and Lipid; ALLHAT-BP, DOX, LLT; WHI Estrogen and Estrogen-Progestin, and WHS aspirin and vitamin E). A total of 55 trials were analyzed– 30 were published prior to 2000 and 25 were published in 2000 or later (see S2 Table for list of included trials. A complete list of the references also appears in the Supplemental Materials).

**Fig 1:**
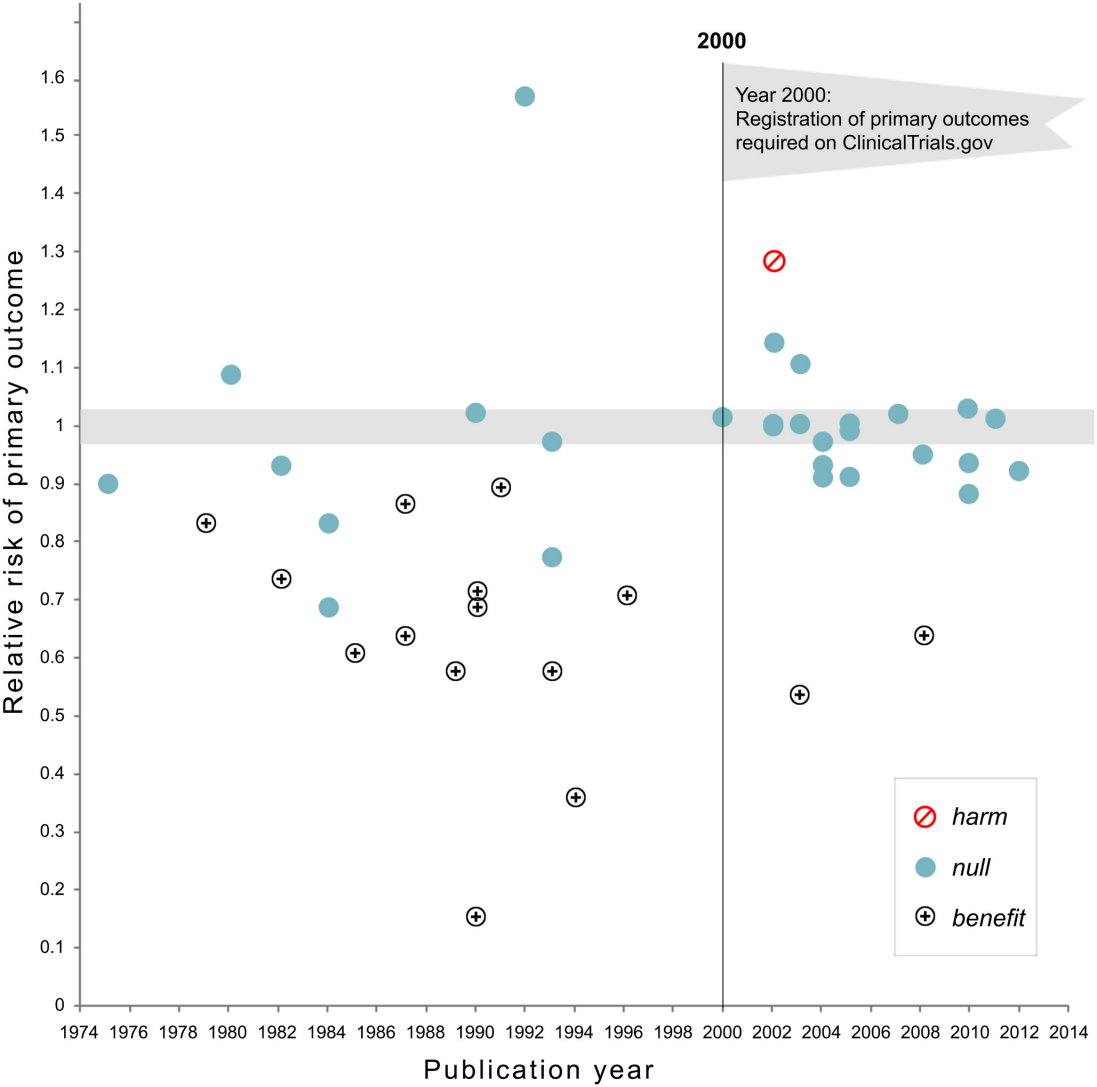
Relative risk of showing benefit or harm of treatment by year of publication for large NHLBI trials on pharmaceutical and dietary supplement interventions. Positive trials are indicated by the plus signs while trials showing harm are indicated by a diagonal line within a circle. Prior to 2000 when trials were not registered in clinical trials.gov, there was substantial variability in outcome. Following the imposition of the requirement that trials preregister in clinical trials.gov the relative risk on primary outcomes showed considerably less variability around 1.0.

**Fig 2:**
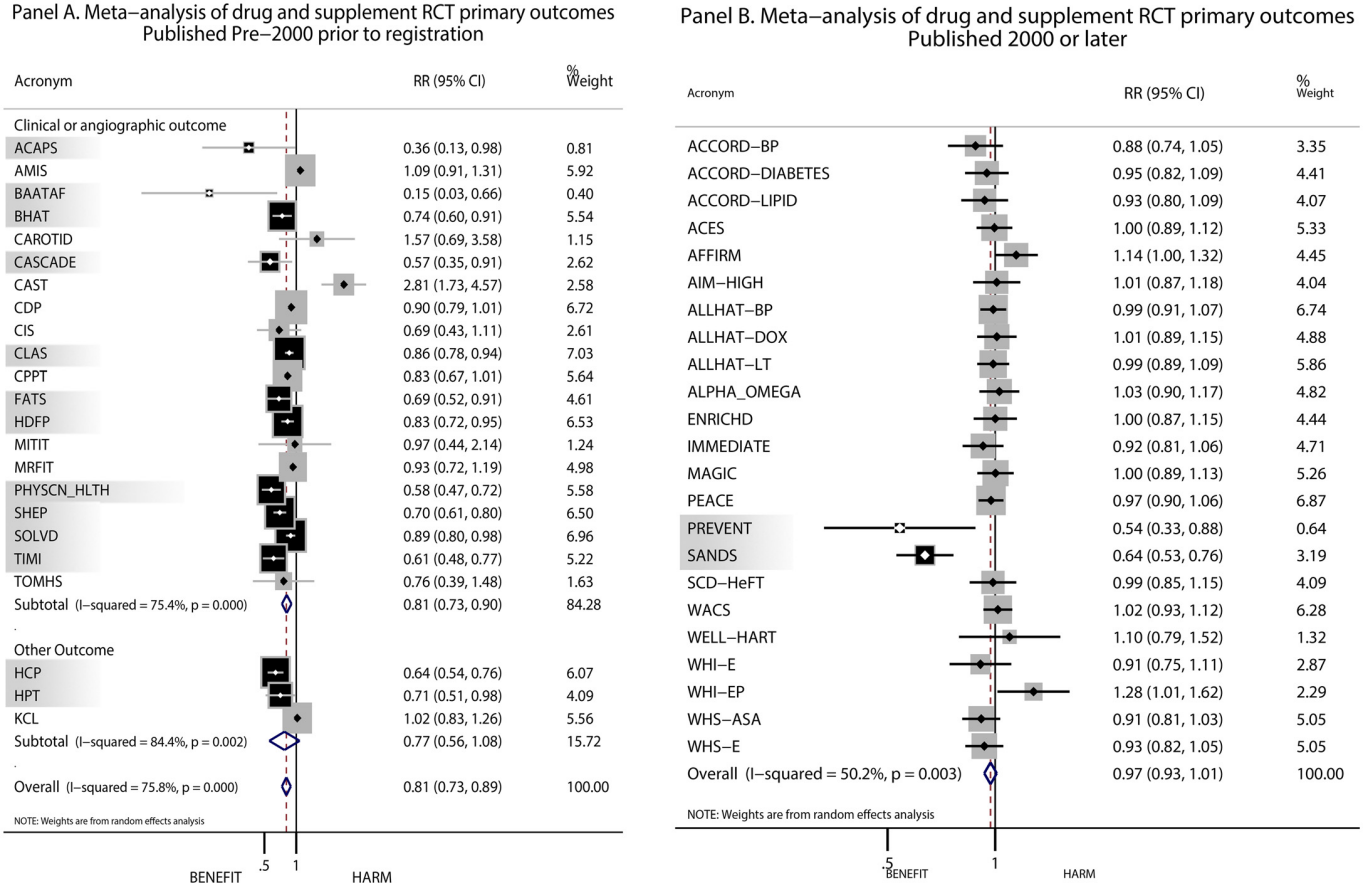
Summary of results on the primary outcome in NHLBI trials on pharmaceutical and supplement interventions that were not pre-registered in clinical trials.gov (panel A) and pre-registered in clinical trials.gov (panel B). Trials indicated by shading and black boxes had statistically significant effects of intervention while trials not shaded and represented by gray boxes had null effects.

Fig 1 plots the relative risks of the primary outcome by the publication year of the main outcome paper. Because it was an extreme outlier, the CAST study is excluded from the figure. Prior to publication in 2000, studies often showed benefits of treatments with the notable exception of CAST (not shown in figure). Following 2000, confidence intervals for relative risk ratios included 1.0 in all cases, with the exceptions of the PREVENT and the SANDS trials (benefit) and the Women’s Health Initiative (Harm). In addition, the variability in RRs was considerably reduced after the year 2000 (Fig 2).

Results for all cause mortality were similar. Prior to 2000, 24 trials reported all cause-mortality and 5 reported significant reductions in total mortality (25%), 18 were null (71%) and one (CAST) reported significant harm (Table 3). Following the year 2000, no study showed a significant benefit for total mortality. An expanded presentation of the results in given in the online supplemental materials, including a figure summarizing results for all cause mortality.

**Table 3:** Summary of Published Drug and Supplement NHLBI Trials, 1970–2012.

	Published Pre-2000-Not Registered	Published Post-2000-Preregistered	χ^2^ for Difference in Benefit, df -1
Number of Trials	30	25	
Primary outcome specified in manuscript	23	25	4.75 p = .029^d^
Consort-like diagram	5	14	9.22 p = .0024^d^
Control Groups			001 p = .9798^d^
Placebo	18	16	
Usual Care	9	7	
Active Comparator	3	2	
Primary Outcome			
Benefit	17	2	12.2, p = 0.0005
Harm	1	1	
Null	12	22	
Mortality			
Benefit	5	1 ^b^	1.14 p = 0.286
Harm	1	2	
Null	13^a^	22^c^	
Not powered	5	0	
Not reported	6	0	

^a.^ The CDP trial did not show mortality benefits with the original follow-up period. If we had analyzed the subsequent follow-up trial that was not apriori, treatment with niacin would have shown a significant benefit to total mortality.

^b.^ The PREVENT study was stopped early for effectiveness seen in the primary outcome. The number of deaths reported shows a null effect on total mortality; but this study might have shown a benefit for total mortality had the full follow-up been completed. We report it here as significant but it will show as null in the meta-analyses.

^c.^ The SANDS study did not show any differences on total mortality or reduction of cardiovascular disease events. However, the treatment arm experienced significantly more adverse events. SANDS study authors concluded that there may not be favorable long-term outcomes for participants randomized to treatment.

^d.^ Chi-square test uses the Yates correction for continuity.

We considered a variety of explanations for the trend toward null results that emerged around 2000. (Detailed tables are given in online supplemental materials). One possibility is that more recent trials may have evaluated their treatment drug against clinically effective alternatives instead of placebos. We do not find this suggestion likely because 60% of the large NHLBI trials published prior to 2000 used a placebo as the comparator in contrast to 64% trials published after 2000 (see S3 Table). Placebos were used as the comparator at about the same rate prior to and after the year 2000 (p = .979).

To investigate the effect of industry co-sponsorship, we tabulated sponsorship for all reports. Unfortunately, industry co-sponsorship was not always reported prior to the year 2000 and journals did not uniformly require disclosure. After the year 2000, when the International Committee of Medical Journal Editors (ICMJE) asked for disclosure, it became apparent that industry co-sponsorship is very common. In our sample, 23 of 25 (92%) of the NHLBI trials published after 2000 had partial industry sponsorship or contribution of medications. All but two of these trials obtained null results. We also looked at previous financial relationships between investigators and industry. Prior to 2000, these relationships were reported in only 1 of the 30 trials (3%). Even after 2000, 28% of the studies did not include a disclosure section. But among articles that included disclosures, there was a financial consulting relationship between at least one author and industry in all (100%) of the cases. Industry influence would produce a bias in favor of positive results, so connections between investigators and industry is not a likely explanation for the trend toward null results in recent years.

We considered a variety of aspects of transparent reporting. Prior to 2000, 5 of the 30 published trials (17%) included a diagram that clearly accounted for the number of participants at each phase of the project. Following 2000, publications were significantly more likely to account for patients throughout the study: 14 of the 25 trials (56%) included such a flow diagram (χ2 = 9.22, p = 0.002). After the year 2000 all of the published papers clearly identified primary outcome variable, while the primary outcome variable was not specified in 23% of the publications prior to 2000 (χ2 = 4.75, p = 0.03).

A final explanation for the trend toward null reports is that current authors face greater constraints in reporting the results of their studies. In our review, the year 2000 marks the beginning of a natural experiment. After the year 2000, all (100%) of large NHLBI were registered prospectively in ClinicalTrials.Gov prior to publication. Prior to 2000 none of the trials (0%) were prospectively registered. Although many of the earlier studies are in the ClinicalTrials.Gov database, they were registered after the results had been published. Following the implementation of ClinicalTrials.gov, investigators were required to prospectively declare their primary and secondary outcome variables. Prior to 2000, investigators had a greater opportunity to measure a range of variables and to select the most successful outcomes when reporting their results. For trials published before the year 2000, we found that 17 out of 30 (57%) reported significant benefit for their primary outcome. In the new era where primary outcomes are prospectively declared (published post 2000), only 2 of 25 trials (8%) reported a significant benefit (χ2 = 12.2, p = 0.0005).

Prospective declaration of the primary outcome variable is important because it eliminates the possibility of selecting for reporting an outcome among many different measures included in the study. In order to investigate this issue, we looked at the statistical significance of other variables not declared as the primary outcomes for preregistered studies. Among the 25 preregistered trials published in 2000 or later, 12 reported significant, positive effects for cardiovascular-related variables other than the primary outcome. Importantly, almost half of the trials might have been able to report a positive result if they had not declared a primary outcome in advance. Had the prospective declaration of a primary outcome not have been required, it is possible that the number of positive studies post-2000 would have looked very similar to the pre-2000 period.

## Discussion

Beginning in approximately 2000, the likelihood of showing a significant benefit in large NHLBI funded studies declined. Among the explanations we evaluated, the requirement of prospective registration in Clinicaltrials.gov is most strongly associated with the observed trend toward null clinical trials. The decline is not easily explained by the increased use of active comparators or a decline in industry sponsorship. In addition to the explanations at we evaluated using reported characteristics of the trials, we considered several other suggestions.

One explanation is that newer clinical trial management methodologies remove error variance and provide more precise estimates of treatment effects. If this were the explanation, refined methodologies and greater precision should have resulted in reductions in error variance, ultimately increasing the likelihood of finding treatments effects. But the probability of finding a treatment benefit decreased rather than increased as studies became more precise. As shown in Fig 1, variability in trial results declined systematically around the year 2000. As a result, we do not find better trial management to be a compelling explanation for the trend toward null results.

It is widely noted that journals favor publication of statistically significant findings[2]. Bias in favor of publishing positive outcomes is not a likely explanation for our results. We focused on large trials because previous analyses by NHLBI reported that 97% of trials with annual budgets over $500,000/year were published [3], thus removing publication bias as a rival explanation. In our analysis, 88% of the trials were published, although there may be a slight delay in the date of publication for null trials^6^. If positive trials are more likely to be published than null trials, we would have expected more positive published reports following 2000. A “file drawer” problem of suppressing null trial findings would result in over reporting positive results. Our observation of a trend toward null results goes in the opposite direction. If there is a bias, it is possible that stricter reporting standards and greater rigor in reporting requirements are suppressing the declaration of positive outcomes.

It has been argued that there have been few efficacious drugs in the pipeline[4,5]. Since about 1998, there has been a systematic decline in the number approvals for new cardiovascular drugs[6]. Thus, we would expect more null trials because the rate of developing effective new principals has declined. We believe this explanation unlikely because nearly all of the trials evaluated treatments that had been previously studied. For example, all of the treatments had been approved by the US FDA and these approvals require early phase trial evidence of safety and efficacy.

Another explanation for the increase in null trials is the possibility that medical care and supportive therapy have improved since 2000. As a result it has become difficult to demonstrate treatment effects because new approaches must compete with higher quality medical care. In support of this argument is the observation that outcomes in cardiovascular diseases continue to improve despite wide variation in the specific care that patients receive. On the other hand, outcomes of studies that compared treatment to an active standard of care comparison group achieved results quite similar to studies that compared treatment to placebo. However, we do recognize that the quality of background cardiovascular care continues to improve, making it increasingly difficult to demonstrate the incremental value of new treatments. The improvement in usual cardiovascular care could serve as alternative explanation for the trend toward null results in recent years.

Our results may also reflect greater involvement by NHLBI in trial design and execution. Prior to 2000, most large NHLBI clinical trials were investigator initiatLaed while nearly 80% of the trials published after 2000 had direct involvement of NHLBI through cooperative agreements. We recognize that industry sponsored trials may have a higher success rate. It is possible that industry conducts trials designed to demonstrate effectiveness while NHLBI uses its resources when there is true equipoise.

All post 2000 trials reported total mortality while total mortality was only reported in about 80% of the pre-2000 trials and many of the early trials were not powered to detect changes in mortality. The effects on total mortality were null for both pooled analyses of trials that were registered or not registered prior to publication (see data in online supplement) In addition, prior to 2000 and the implementation of Clinicaltrials.gov, investigators had the opportunity to change the p level or the directionality of their hypothesis *post hoc*. Further, they could create composite variables by adding variables together in a way that favored their hypothesis. Preregistration in ClinicalTrials.gov essentially eliminated this possibility.

### Limitations

Our analysis is limited to large NHLBI-funded trials and to studies on cardiovascular outcomes in adults. We focused on NHLBI because the Institute has championed transparency and allowed us full access to all trials. We emphasized large trials because we had access to outcomes of nearly all studies, thus reducing the risk of publication bias. Although we focused on cardiovascular trials, null results are common in other areas of medicine. For example, among 221 agents with the potential to modify outcomes for Alzheimer’s disease, all placebo controlled trials registered in clinical trials.gov have failed to identify positive benefits on the declared primary outcome[7].

Our analysis underscores the importance of NHLBI involvement in trials. A greater number of recent trials used direct NHLBI, oversight. The institute is fully vetted for conflict of interest and applies high quality control standards including full transparency, open data access, and registration in ClincalTrials.gov. Our conclusions may not generalize to trials sponsored by industry or to other funding agencies.

We cannot say that trend toward null trials to preregistration in ClinicalTrials.gov is causal. Our analysis included only a small number of trials and the design of the study does not allow causal inferences. Most importantly, many variables may have changed around the year 2000. It is likely that other variables that are unknown or unmeasured also correspond to the decline in reports of significant therapeutic treatment effects.

### Implications

The transparency of RCTs is likely to have improved following the FDA Modernization Act of 1997, which created the ClinicalTrials.gov registry[8], a service that required registration of studies that test drugs, biologics, or devices for the treatment of serious or life threatening diseases[9–11]. Registered studies must provide: the study’s purpose, recruitment status, design, eligibility criteria, locations and pre-specified primary and secondary outcomes[11]. The Consolidated Standards of Reporting Trials (CONSORT) were introduced in 1996 but expanded in 2001 to require greater transparency in the reporting of Randomized Clinical Trials (RCTs)[12]. Shortly after 2001, many major journals began requiring prospective registration of clinical trials as a condition for publication and the International Committee of Medical Journal Editors started requiring CONSORT reporting in all major journals beginning in 2004 (icjme.org). NHLBI was an early adopter of trial registration. All of their large trials published after 2000 were preregistered and transparently reported. Although we cannot say that stricter reporting requirements caused the trend toward more null reports from NHLBI trials, we do find the association worthy of more investigation.

In conclusion, null findings in large RCTs may be disappointing to investigators, but they are not negative for science. Properly powered trials might identify treatments that will improve public health. A growing collection of trials suggests that promising treatments do not match their potential when systematically tested and transparently reported. Publication of these trials may lead to the protection of patients from treatments that use resources while not enhancing patient outcomes. For example, a recent economic analysis of the Women’s Health Initiative clinical trial suggested that the publication of the study may have resulted in 126,000 fewer breast cancer deaths, and 76,000 deaths from heart disease between 2003 and 2012. The economic analysis estimated that there was about $140 returned for each dollar invested in the study[13]. Transparent and impartial reporting of clinical trial results will ultimately identify the treatments most likely to maximize benefit and reduce harm.

## Supporting Information

S1 Fig PRISMA Diagram. (TIFF)

S2 Fig Summary of results on all cause mortality. (TIF)

S1 Table Total number of returned searches in NIH grant databases, type and number of exclusions, number of grants identified, and total number of trials analyzed. (PDF)

S2 Table Sample sizes, number of primary outcome events, number of deaths from all causes for trials published before the year 2000. (PDF)

S3 Table Sample sizes, number of primary outcome events, number of deaths from all causes for trials published in the year 2000 or later. (PDF)

S4 Table Expanded Table 1. (PDF)

S5 Table Expanded Table 2. (DOCX)

S1 Text Expanded Methods Section. (PDF)

S2 Text Expanded Results Section. (PDF)

S3 Text List of Acronyms. (PDF)

S4 Text List of Studies Included in Meta Analysis. (PDF)
