# Supplementary figures and images for: Likelihood of Null Effects of Large NHLBI Clinical Trials Has Increased over Time
Source: PLoS One. 2015 Aug 5;10(8):e0132382. doi: 10.1371/journal.pone.0132382 (PMC4526697; doi:10.1371/journal.pone.0132382)

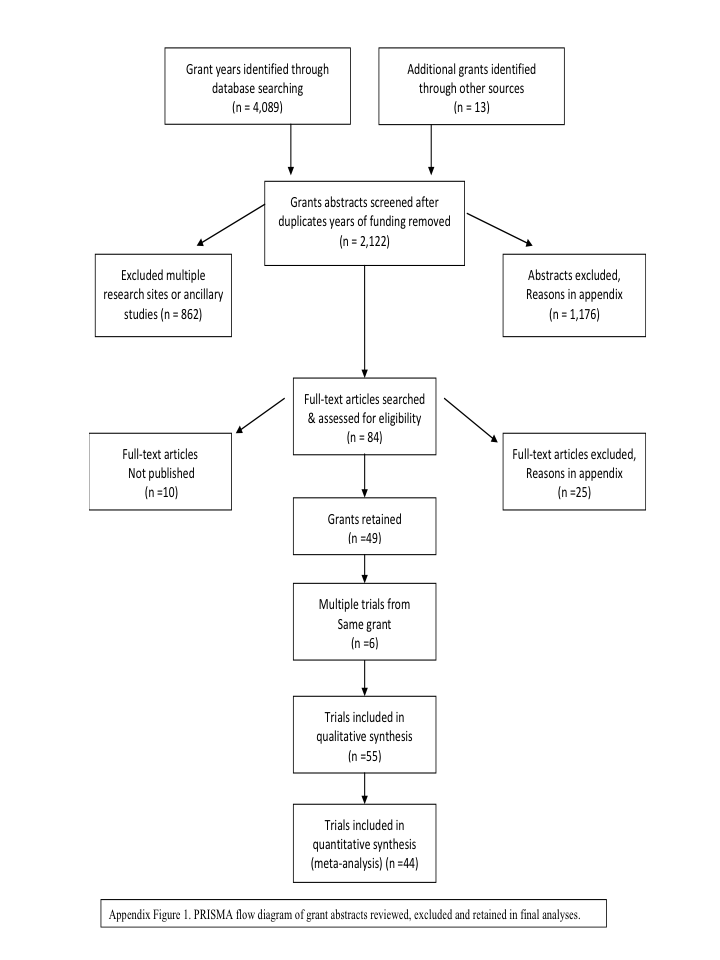

Supplement: S1 Fig — (TIFF) [file pone.0132382.s001.tiff]

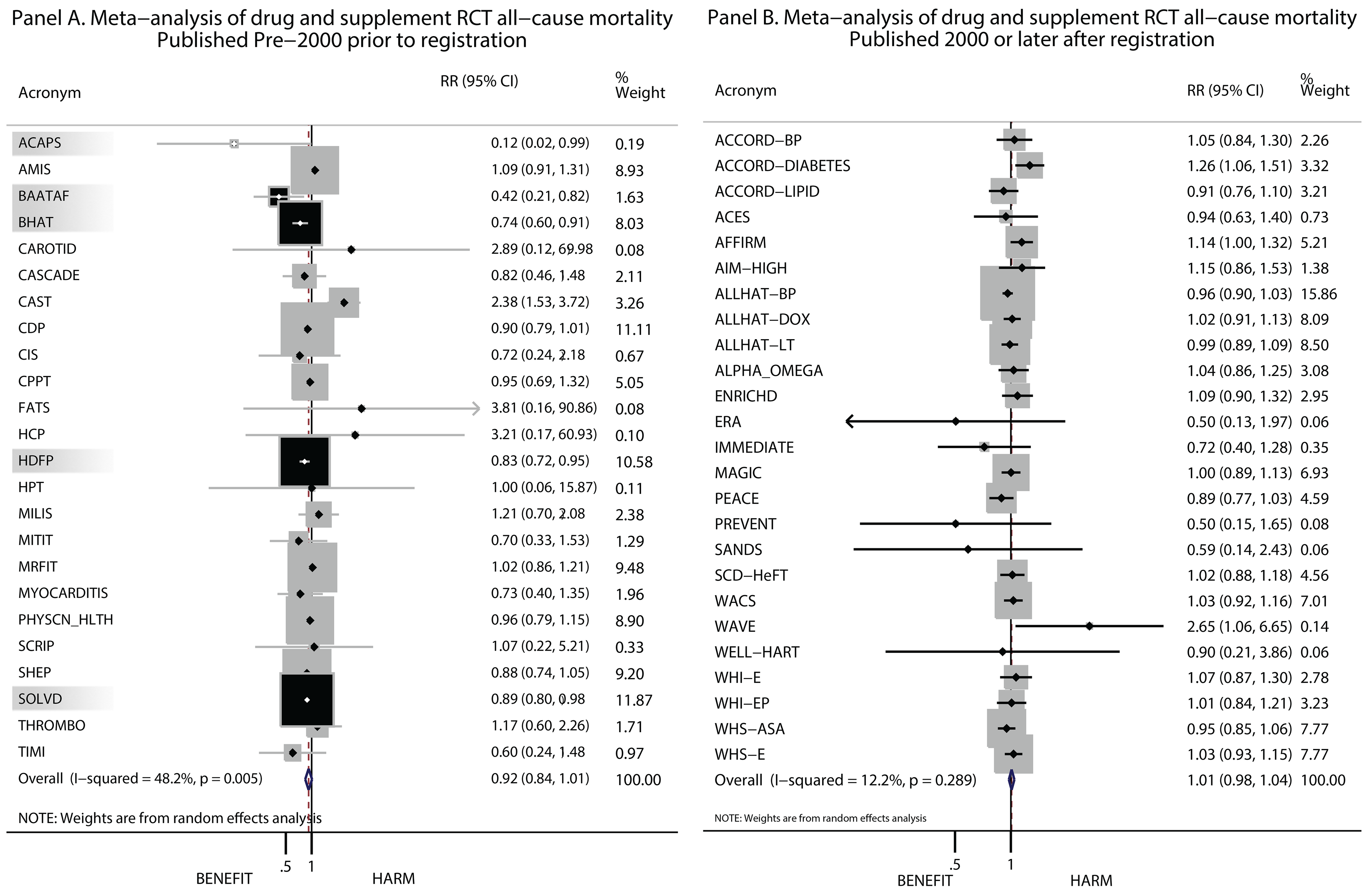

Supplement: S2 Fig — (TIF) [file pone.0132382.s002.tif]
