# Supplementary material for: Likelihood of Null Effects of Large NHLBI Clinical Trials Has Increased over Time
Source: PLoS One. 2015 Aug 5;10(8):e0132382. doi: 10.1371/journal.pone.0132382 (PMC4526697; doi:10.1371/journal.pone.0132382)
Supplement: S1 Table — (PDF) [file pone.0132382.s003.pdf]

Appendix Table 1. Total number of returned searches in NIH grant databases, type and number of exclusions, number of grants identified, and total number of trials analyzed	
	Number
Total grant years retrieved from searches from NIH grant databases[a]	4089
Additional grants found in NHLBI search	13
Duplicate years of funding removed from same grant	1,980
Number of grant abstracts reviewed after removing duplicate years of funding	2,122
Duplicate sites, ancillary studies or coordinating centers from same grant	862
Abstracts excluded after review, reasons below	1,176
K or T award	40
No human subject approval required	112
Only children included	90
No human participants; only biospecimens, etc	17
Animal	11
Lung related	114
Blood, circulatory related	135
Genetics, gene therapy, genotype guided	39
Not randomized clinical trial	173
Active grant	245
Kidney related	1
Stem cells related	9
Technology/measurement development	46
Hypo/hyperthermia related	2
Cell therapy related	3
Behavior intervention/surveillance	61
Community trial	12
No Cardiovascular outcome	12
Pilot, feasibility studies	6
Imaging only studies	3
Registries, networks, repositories	14
Service, delivery	4
Surgery	64
Media	14
Abstract aimed to analyze only sub-groups	2
Transplants	2
Device/procedures	1
Full-text articles searched and reviewed	84
Articles not published	10
Articles excluded, reasons below	25
Behavior intervention/surveillance	7
Biologic	1
Device/procedure	7
Trial stopped early	1
Surgery	6
No Cardiovascular outcome	1
Grant with published outcome retained	49
Multiple "trials" from same grant (ACCORD, ALL HAT, WHI, WHS)	6
Final number of trials analyzed	55
a. NIH grant databases searched were QVR, Reporter and CRISP	
