# Supplementary material for: Likelihood of Null Effects of Large NHLBI Clinical Trials Has Increased over Time
Source: PLoS One. 2015 Aug 5;10(8):e0132382. doi: 10.1371/journal.pone.0132382 (PMC4526697; doi:10.1371/journal.pone.0132382)
Supplement: S2 Table — (PDF) [file pone.0132382.s004.pdf]

Appendix Table 2. Sample sizes, number of primary outcome events, number of deaths from all causes for trials published before the year 2000.								
Acronym	Treatment N size	Control N Size	Treatment Events	Control Events	Treatment Deaths	Control Deaths	Primary Outcome	Total mortality
ACAPS[a]	460	459	5	14	1	8	BENEFIT	BENEFIT
AMIS	2267	2257	218	199	218	199	NULL	NULL
BAATAF	212	208	2	13	11	26	BENEFIT	BENEFIT
BHAT	1916	1921	138	188	138	188	BENEFIT	BENEFIT
Carotid	83	80	13	8	1	0	NULL	NP
CASCADE	113	115	20	36	17	21	BENEFIT	NULL
CAST	755	743	60	21	63	26	HARM	HARM
CDP	1119	2789	273	709	273	709	NULL	NULL
CIS	71	72	19	28	5	7	NULL	NULL
CLAS[b]	94	94	79	92	NR	NR	BENEFIT	NR
CPPT	1906	1900	155	187	68	71	NULL	NULL
FATS[b]	36	46	22	41	1	0	BENEFIT	NP
FEN-PHEN	62	59	CO	CO	NR	NR	BENEFIT	NR
FISH OIL	9	9	CO	CO	NR	NR	NULL	NR
HCP[b]	97	44	59	42	3	0	BENEFIT	NP
HDFP	5485	5455	349	419	349	419	BENEFIT	BENEFIT
HPT	196	196	46	65	1	1	BENEFIT	NP
MILIS	134	135	CO	CO	24	20	NULL	NULL
MITIT	175	185	CO	CO	10	15	NULL	NULL
MRFIT	6428	6438	115	124	265	260	NULL	NULL
MYOCARDITIS	64	47	CO	CO	15	15	NULL	NULL
KCL	142	145	79	79	NR	NR	NULL	NR
PEPI	701	174	CO	CO	NR	NR	BENEFIT	NR
Physicians Health Study	11037	11034	139	239	217	227	BENEFIT	NULL
SCRIP	145	155	CO	CO	3	3	BENEFIT	NULL
SHEP	2365	2371	288	413	212	241	BENEFIT	NULL
SOLVD	1285	1284	452	510	452	510	BENEFIT	BENEFIT
TIMI-1[b]	143	147	57	96	7	12	BENEFIT	NP
THROMBO	95	97	CO	CO	16	14	BENEFIT	NULL
TOMHS	668	234	26	12	NR	NR	NULL	NR

Notes: CO = Primary outcome was continuous and excluded from meta-analysis. NR = Not reported. NP = Not powered. a = Primary outcome was continuous but substituted binary outcome to run in meta-analysis; both original and substituted primary outcome had same end result. For ACAPS, primary outcome reported was 3-year change in IMT wall of carotid artery (sig differences $p<.001$), but we analyzed secondary outcomes of cardiovascular events which were both significant (5 for treatment, 14 for placebo, $p=.04$). b= Primary outcome had to be reverse coded in order to match the direction of the other primary outcomes.
