# Supplementary material for: Likelihood of Null Effects of Large NHLBI Clinical Trials Has Increased over Time
Source: PLoS One. 2015 Aug 5;10(8):e0132382. doi: 10.1371/journal.pone.0132382 (PMC4526697; doi:10.1371/journal.pone.0132382)
Supplement: S3 Table — (PDF) [file pone.0132382.s005.pdf]

Appendix Table 3. Sample sizes, number of primary outcome events, number of deaths from all causes for trials published in the year 2000 or later.								
Acronym	Treatment N size	Control N Size	Treatment Events	Control Events	Treatment Deaths	Control Deaths	Primary Outcome	Total mortality
ACCORD-BP	2362	2371	208	237	150	144	NULL	NULL
ACCORD-Diabetes	5128	5123	352	371	257	203	NULL	HARM
Accord-Lipid	2765	2753	291	310	203	221	NULL	NULL
ACES	2004	2008	446	449	143	132	NULL	NULL
AFFIRM	2033	2027	356	310	356	310	NULL	NULL
AIM-HIGH	1718	1696	282	274	96	82	NULL	NULL
ALLHAT-BP	9048	15255	798	1362	1256	2203	NULL	NULL
ALLHAT-LT	5170	5185	631	641	631	641	NULL	NULL
ALLHAT-DOX	9067	15268	365	608	514	851	NULL	NULL
Alpha Omega	2404	2433	336	335	186	184	NULL	NULL
ENRICHD	1238	1243	299	300	168	172	NULL	NULL
ERA[a]	104	105	CO	CO	3	6	NULL	NULL
IMMEDIATE	411	460	200	242	18	28	NULL	NULL
MAGIC	3113	3100	475	472	475	472	NULL	NULL
PEACE	4158	4132	909	929	299	334	NULL	NULL
PREVENT[b]	255	253	22	41	4	8	BENEFIT	NULL
SANDS[a]	252	247	98	151	3	5	BENEFIT	NULL
SCD-HeFT	845	847	240	244	240	244	NULL	NULL
WACS	4084	4087	731	719	505	490	NULL	NULL
WAVE[a,c]	212	211	CO	CO	16	6	NULL	HARM
WELL-HART[a]	54	61	31	34	3	4	NULL	NULL
WHI-EP	8506	8102	164	122	231	218	HARM	NULL
WHI-E	5310	5429	177	199	291	289	NULL	NULL
WHS-ASA	19934	19942	477	522	609	642	NULL	NULL
WHS-E	19937	19939	482	517	636	615	NULL	NULL
Notes: CO = Primary outcome was continuous and excluded from meta-analysis. a= Primary outcome was continuous but substituted binary outcome to run in meta-analysis; both original and substituted primary outcome had same end result. For WELL-HART primary outcome was change in percent stenosis; we analyzed number of participant with progression of artery; both were null. Primary outcome for SANDS trial was intimal median thickness of the carotid artery which was continuous. We substituted the binary outcome percent of participants who experienced an increase in the CIMT at follow-up. Both binary and continuous outcomes were significantly better in treatment group than control at follow-up.								

For ERA and WAVE, primary outcomes were angiographic- mean minimal coronary artery diameter; no binary outcomes available. b= Trial stopped early because of effectiveness in primary outcome; if trial had completed original follow-up total mortality may have been significant. c=WAVE trial had two different arms – estrogen vs placebo or vitamin E and C vs placebo. We reported the effects of the Vitamin E & C arms. For the estrogen arm, primary outcome of coronary arm diameter was null and total mortality was null but had the potential for harm.
