# Supplementary material for: Likelihood of Null Effects of Large NHLBI Clinical Trials Has Increased over Time
Source: PLoS One. 2015 Aug 5;10(8):e0132382. doi: 10.1371/journal.pone.0132382 (PMC4526697; doi:10.1371/journal.pone.0132382)
Supplement: S4 Table — (PDF) [file pone.0132382.s006.pdf]

Expanded Table 1

Table 1. Study characteristics and overall effect for main outcome and total mortality for studies not registered in ClinicalTrials.gov prior to publication

Acronym	Start Year	Pub Year	Drug	Primary Outcome (PO)	Registry #	Date First Registered	PO specified	Contract or Cooperative Agreement	Comparator	Consort	Industry Funding	Financial Disclosures	Primary Outcome	Total mortality
ACAPS	1988	1994	Lovastatin	3-year change in IMT wall of CA	NCT469	1999	Yes	No	Placebo	No	M & D	NR	Benefit	Benefit
AMIS	1974	1980	Apsirin	All-cause mortality	NCT491	1999	Yes	Yes	Placebo	No	No	NR	Null	Null
BAATAF	1985	1990	Warfarin	Prevalence of stroke	NCT517	1999	Yes	No	Aspirin OR placebo	No	No	NR	Benefit	Benefit
BHAT	1977	1982	Propanol	All-cause mortality	NCT492	1999	Yes	Yes	Placebo	No	D	NR	Benefit	Benefit
Carotid	1986	1992	Aspirin & dipyridamole	>50% restenosis at 1 year	NCT527	1999	No	No	Placebo	No	NR	NR	Null	NP
CASCADE	1987	1993	Amiodarone	Composite of survival free of various cardiac events	NCT464	1999	Yes	No	Conventional therapy	No	No	NR	Benefit	Null
CAST	1986	1991	Antiarrhythmic drugs	Cardiac death or arrest	NCT526	1999	Yes	Yes	Placebo	No	No	NR	Harm	Harm
CDP	1965	1975	Niacin	All-cause mortality	NCT482	1999	Yes	Yes	Placebo	Yes	No	NR	Null	Null
CIS	1971	1984	Cholestyramine	Progression of CAD	NCT594	1999	No	Yes	Placebo	No	No	NR	Null	Null
CLAS	1979	1987	Colestipol or niacin	Atherosclerosis regression	NCT599	1999	No	No	Placebo	Yes	No	NR	Benefit	NR

CPPT	1971	1984	Cholestyramine	CHD death or definite non-fatal MI	NCT488	1999	Yes	Yes	Placebo	No	No	NR	Null	Null
FATS	1984	1990	Lovastatin & colestipol	Regression primary artery	NCT512	1999	No	No	Conventional therapy plus placebo, if cholesterol high given lovastin	No	D	NR	Benefit	NP
FEN-PHEN	1983	1992	Fen phen	Weight loss	NCT506	1999	Yes	No	Placebo	No	NR	NR	Benefit	NR
FISH OIL	1983	1993	Fish oil	Blood pressure	NCT461	1999	Yes	No	6 VS 12 GRAMS	No	No	NR	Null	NR
HCP	1980	1987	Antihypertensive agents & behavior change	Normative bp	NCT498	1999	Yes	No	Continued drug therapy	Yes	M & D	NR	Benefit	NP
HDFP	1971	1979	Anti-hypertensives	All-cause mortality	NCT485	1999	Yes	Yes	referred care	No	D	NR	Benefit	Benefit
HPT	1981	1990	Na & K and behavior change	Composite of on medication or dbp>90 sbp>140	NCT501	1999	Yes	No	no counseling	No	No	NR	Benefit	NP
KCL	1983	1990	KCL	Reinstated blood pressure med	NCT509	1999	Yes	No	Placebo	No	No	NR	Null	NR
MILIS	1977	1984	Propanol	Infarct size	NCT493	1999	No	Yes	Placebo	No	No	NR	Null	Null
MITIT	1988	1993	Aspirin and atleplase before arrival at hospital	Ranked composite of death, stroke, bleeding, infarct size	NCT468	1999	Yes	No	Nothing at first, but same given after hospital	Yes	M	NR	Null	Null
MRFIT	1974	1982	Stepped care	CHD death	NCT112	1999	Yes	Yes	Usual care	No	D	NR	Null	Null

					06									
MYOCARDITIS	1986	1995	Cyclosporine, prednisone	Change in left ventricular infarction size	NCT524	1999	Yes	No	Conventional therapy	No	No	NR	Null	Null
PEPI	1994	1995	Estrogen + medroxyprogresterone	Lipoproteins, blood pressure	NCT466	1999	Yes	Yes	placebo	No	No	NR	Benefit	NR
Physicians Health Study	1981	1989	Aspirin	MI infarction	NCT500	1999	Yes	No	Placebo	No	No	NR	Benefit	Null
SCRIP	1983	1994	Meds & behavior	Angiographic change of diameter	NCT508	1999	No	No	Usual care	Yes	D	NR	Benefit	Null
SHEP	1984	1996	Chlorthalidone	Major cvd events	NCT514	1999	No	Yes	Placebo	No	D	NR	Benefit	Null
SOLVD	1975	1991	Enalapril	All-cause mortality	NCT516	1999	Yes	Yes	Placebo	No	M	NR	Benefit	Benefit
Thrombo	1982	1989	Nitroglycerin + streptokinase	Change in angiographic endpoint ejection fraction	NCT503	1999	Yes	No	nitroglyceride alone	No	D	NR	Benefit	Null
TIMI	1983	1985	Tissue plasminogen	Reperfusion	NCT505	1999	Yes	Yes	Alternate tx	No	No	CF	Benefit	NP
TOMHS	1985	1993	Anti-hypertensives	Major cvd events	NCT522	1999	Yes	No	Placebo	No	M & D	NR	Null	NR

NR= Not reported. NP = Not powered., CF = Consulting Fees; M = Industry contributed some money; D = Industry provided pharmaceutical drugs
