# Supplementary material for: Likelihood of Null Effects of Large NHLBI Clinical Trials Has Increased over Time
Source: PLoS One. 2015 Aug 5;10(8):e0132382. doi: 10.1371/journal.pone.0132382 (PMC4526697; doi:10.1371/journal.pone.0132382)
Supplement: S5 Table — (DOCX) [file pone.0132382.s007.docx]

Expanded Table 2

| Table 2. Study characteristics and overall effect for main outcome and total mortality for studies registered in ClinicalTrials.gov prior to publication | | | | | | | | | | | | | | |
| --- | --- | --- | --- | --- | --- | --- | --- | --- | --- | --- | --- | --- | --- | --- |
| Acronym | Start Year | Pub Year | Drug | Primary Outcome (PO) | Registry # | Date First Registered | PO specified | Contract or Cooperative Agreement | Comparator | Consort | Industry Funding | Financial Disclosures | Primary Outcome | Total mortality |
| ACCORD-BP | 2000 | 2010 | Bld press control meds | Composite - Non-fatal MI, non-fatal stroke, CVD death | NCT620 | 1999 | Yes | Yes | standard therapy | No | D | NR | Null | Null |
| ACCORD-Diabetes | 2000 | 2008 | Intensive tx to target normal glycated hemoglobin levels | Major or non-fatal MI, non-fatal stroke, CVD death | NCT620 | 1999 | Yes | Yes | standard therapy | No | D | CF | Null | Harm |
| Accord-Lipid | 2000 | 2010 | Simvastatin  +  fenofibrate | Major non fatal MI stroke or CVD death | NCT620 | 1999 | Yes | Yes | standard therapy (simvastatin) | No | D | CF | Null | Null |
| ACES | 1998 | 2005 | Azithromycin | Composite - death from CV,revacularization,hospitalization | NCT617 | 1999 | Yes | Yes | Placebo | No | M | CF | Null | Null |
| AFFIRM | 1995 | 2002 | Anti-arrhythmic | All-cause mortality | NCT556 | 1999 | Yes | Yes | controlled regulation | No | D | CF | Null | Null |
| AIM-HIGH | 2005 | 2011 | Niacin + statin | composite death plus events | NCT120289 | 2005 | Yes | Yes | Placebo | No | M & D | CF | Null | Null |
| ALLHAT-BP | 1993 | 2002 | Amlodipine | Fatal or non-fatal MI | NCT542 | 1999 | Yes | Yes | Active comparator (diuretic) | Yes | M & D | CF | Null | Null |
| ALLHAT-DOX | 1993 | 2000 | Doxazosin | Fatal or non-fatal MI | NCT542 | 1999 | Yes | Yes | Active comparator | Yes | M & D | CF | Null | Null |
| ALLHAT-LLT | 1993 | 2002 | Pravastatin | All cause mortality | NCT542 | 1999 | Yes | Yes | Usual care | Yes | M & D | CF | Null | Null |
| Alpha Omega | 2005 | 2010 | Omega-3 Fatty Acids | Fatal and nonfatal cardiovascular | NCT127452 | 2005 | Yes | No | Placebo | Yes | D | NR | Null | Null |
| ENRICHD | 1995 | 2003 | Anti-depressants & CBT | Death or recurrent MI | NCT557 | 1999 | Yes | Yes | Usual care | Yes | D | NR | Null | Null |
| ERA | 1994 | 2000 | Estrogen + medroxyprogresterone | Mean minimal coronary artery diameter | NCT549 | 1999 | Yes | Yes | Placebo | No | D | NR | Null | Null |
| IMMEDIATE | 2004 | 2012 | Drug intravenous glucose-insulin-potassium | Progression of ACS to MI | NCT91507 | 2004 | Yes | Yes | Placebo | Yes | D | CF | Null | Null |
| MAGIC | 1998 | 2002 | Magnesium | 30 day all cause mortality | NCT610 | 1999 | Yes | Yes | Placebo | Yes | No | NR | Null | Null |
| PEACE | 1995 | 2004 | Drug -Angiotensin-converting-enzyme (ACE) inhibitors | Death from CVD | NCT558 | 1999 | Yes | Yes | Placebo | No | M & D | CF | Null | Null |
| PREVENT | 1998 | 2003 | Warfarin | Recurrent venous thromboembolism | NCT614 | 1999 | Yes | No | Placebo | No | D | CF | Benefit | Null |
| SANDS | 2002 | 2008 | Aggressive treatment with lipid and blood pressure medications | Carotid artery intimal medial thickness | NCT47424 | 2002 | Yes | Yes | Standard therapy | Yes | D | CF | Benefit | Null |
| SCD-HeFT | 1997 | 2005 | Amiodarone | Total Mortality | NCT609 | 1999 | Yes | Yes | Placebo | No | M | CF | Null | Null |
| WACS | 1993 | 2007 | Beta-carotene | CVD death or events | NCT541 | 1999 | Yes | No | placebo | Yes | D | NR | Null | Null |
| WAVE | 1996 | 2002 | Vitamin E and C | Change in minimum luminal diameter | NCT555 | 1999 | Yes | Yes | Placebo | Yes | No | NR | Null | Harm |
| WELL-HART | 1995 | 2003 | Estradiol+medroxyprogesterone | Change in percent stenosis | NCT559 | 1999 | Yes | Yes | Placebo | Yes | D | CF | Null | Null |
| WHI-E | 1999 | 2004 | Estrogen | CHD Incidence | NCT611 | 1999 | Yes | Yes | placebo | Yes | D | CF | Null | Null |
| WHI-EP | 1999 | 2002 | Estrogen + progestin | CHD Incidence | NCT611 | 1999 | Yes | Yes | Placebo | Yes | D | CF | Harm | Null |
| WHS-ASA | 1991 | 2005 | Aspirin | Non-fatal MI or Stroke or death from CVD | NCT479 | 1999 | Yes | No | Placebo | No | D | CF | Null | Null |
| WHS-E | 1991 | 2005 | Vitamin E | Events and cardiac interventions. | NCT479 | 1999 | Yes | No | placebo | Yes | D | CF | Null | Null |
| NR= Not reported. NP = Not powered.,CF = Consulting Fees; M = Industry contributed some money; D = Industry provided pharmaceutical drugs | | | | | | | | | | | | | | |
