# Supplementary material for: Likelihood of Null Effects of Large NHLBI Clinical Trials Has Increased over Time
Source: PLoS One. 2015 Aug 5;10(8):e0132382. doi: 10.1371/journal.pone.0132382 (PMC4526697; doi:10.1371/journal.pone.0132382)
Supplement: S1 Text — (PDF) [file pone.0132382.s008.pdf]

Supplemental Materials: Expanded Methods Section

Sample of Studies. Our analysis focused on large RCTs that involved drugs or supplements funded between 1970 – 2012. We focused on large trials because NHLBI has shown that virtually all of these studies eventually get published, thus eliminating bias due to selective publication[1]. The search process is summarized in a PRISMA diagram (Apendix Figure 1). Two independent searches were conducted – one by the study authors and the second by NHLBI. We searched three different NIH grant databases (QVR, NIH REPORTER, and CRISP) for RCTs that were primarily funded or administered by NHLBI. QVR is an internal NIH data-base, but readers can replicate our search using NIH REPORTER and CRISP which are publically available resources listing all grants and associated publications. Inclusion criteria were: RCT for studies funded from 1970-2012; grants or contracts; direct costs funded were large enough to require special authorization (>$500,000/ year); the word "trial" had to appear in the study objectives or abstract; and primary outcome was cardiovascular risk factor, event or death. Exclusion criteria included: project still active; no human subjects protocol required; pediatric studies; animal studies; non-RCTs (i.e. observational, cohort, case control, genetic or proteomics, measurement, basic clinical research); or interventions that did not involve a drug or supplement (i.e. behavior change, devices, surgeries).

A second independent search was conducted by NHLBI, Division of Cardiovascular Sciences, to identify clinical trials with budgets requiring special authorization (>$500,000 in any year) funded through 2012[2].

Selection of Outcomes

For each study, we considered the comparison between a treatment and a control condition. Overall, we selected contrasts that gave the treatment the best chance of demonstrating a statistically significant effect. If there were multiple arms in the trial; we analyzed the two groups that were most

different following treatment. For studies that had multiple follow-up periods, we selected the follow-up that showed the strongest treatment effect. Typically, this was the shortest-term follow-up. For the CDP trial, a follow-up measure was added that was not part of the original trial. For this analysis, only the apriori follow-up period was analyzed.

Selection of Primary Outcome

For each study, we identified a primary outcome variable. In the studies preregistered in clinical trials.gov, the papers typically identified the primary outcome (Tables 1 and 2). For publications without an identified primary outcome, we selected a major outcome that was not a sub-analysis. We chose the primary outcome that was in the abstract or in the grant abstract or clinicaltrials.gov documents from NIH. When the information was not available, we attempted to contact investigators. If there were multiple outcome variables, we selected the outcome that was the topic of the article. When authors discussed multiple outcomes, we emphasized the one with the largest treatment effect. If reported, we analyzed total mortality. Sample sizes for populations, number of events and number of deaths for each trial are listed in Appendix Tables 2 and 3.

Independent Variables.

We coded the following variables: start year (earliest funding noted), publication year of main outcome study, funded through contract or cooperative agreement from NHLBI, type of comparator (placebo, active comparator, usual care), consort diagram included in publication, whether funding was exclusively from NIH versus joint industry/NIH funded (including industry contributed medications), and if they had listed any other significant results that were neither the primary outcomes or the side effects of the drug. In addition, we considered whether studies were registered in clinicaltrials.gov prior to publication.

Analysis

Each trial was categorized as showing significant benefit, null, or significant harm for the primary outcome and for total mortality. Null was defined as a confidence interval for the RR that included 1.0.using a two-tailed test. The analysis was standardized by re-computing the relative risk

(RR) with 95% confidence intervals (CI) for all trials. We used a two-tailed alpha of 0.05. Meta-analysis was conducted using STATA-12 using the metan procedure and a random effects model. Meta-analyses were conducted separately for trials that had not preregistered and those that registered prior to data collection. Studies were weighted by the standard error and then rerun weighted by sample size. We did not calculate RR for trials that only provided continuous outcomes (such as weight change), and we did not include these trials in the meta-analyses. However, they are included in Tables 1 and 2 and in the lists of benefit, null or harm trials. This study was determined exempt from review by the National Institute of Health, Office of Human Subjects Research.
