# Supplementary material for: Likelihood of Null Effects of Large NHLBI Clinical Trials Has Increased over Time
Source: PLoS One. 2015 Aug 5;10(8):e0132382. doi: 10.1371/journal.pone.0132382 (PMC4526697; doi:10.1371/journal.pone.0132382)
Supplement: S2 Text — (PDF) [file pone.0132382.s009.pdf]

Supplemental Materials: Expanded Results Section

Our search of the NHLBI databases identified 4,089 individual years of grant funding that met our search criteria. Almost half were excluded as multiple years of the same grant and over 20% were excluded because they were single sites in multi-site trials, coordinating centers, or ancillary studies of the same trial. An additional 1,176 grant abstracts did not match our criteria and were excluded (see Appendix Table 1 for detailed reasons). Main outcome papers were searched for 84 trials; 10 were not published and 25 did not match search criteria and were excluded (See Appendix Figure 1 for the PRISMA diagram and Appendix Table 1 for the number of studies excluded by reason.) Following exclusions, we identified a total of 49 funded grants. Four of these grants resulted in multiple unique trials (ACCORD Blood Pressure, Diabetes, and Lipid; ALLHAT-BP, DOX, LLT; WHI Estrogen and Estrogen-Progestin, and WHS aspirin and vitamin E). A total of 55 trials were analyzed– 30 were published prior to 2000 and 25 were published in 2000 or later (see Appendix Table 2 for list of included trials).

The year 2000 may have been important for NHLBI because it marks the initiation of a policy that required grantees to register trials in clinicaltrials.gov. In this sample, all studies were eventually registered. However, registration of studies in Clinicaltrials.gov prior to publication maps perfectly onto year of publication. No trials published prior to 2000 had pre-registered (0%), while all trials published after 2000 (100%) had registered prior to publication. (See Table 2). Although trials published prior to 2000 also appear in Clinicaltrials.gov, none were registered prior to data collection. Since publication before or after 2000 was perfectly correlated with pre-registration in clinicaltrials.gov, we use these references interchangeably. The subsequent analyses compare publications prior to 2000 to those published 2000 or later.

Figure 1 plots the relative risks of the primary outcome by the publication year of the main outcome paper. Because it was an extreme outlier, the CAST study is excluded from the Figure. Prior to 2000 studies often showed benefits of treatments with the notable exception of CAST (not shown in Figure). Following 2000, confidence intervals for relative risk ratios

included 1.0 in all cases, with the exceptions of the PREVENT and the SANDS trials (benefit) and the Women's Health Initiative (Harm). In addition, the variability in RRs was considerably reduced after 2000.

Tables 1 and 2 list the study characteristics and whether primary outcome and total mortality indicated a significant benefit, null effect or showed harm (Table 1 is for trials published before 2000 and Table 2 for trials published after 2000). Table 3 summarizes comparisons between trials published prior to 2000 and those published after 2000. Of the 30 studies published prior to 2000, 23 (76.7%) specified their primary outcome in the manuscript in comparison to 25 (100%) of trials published after 2000 (Fisher's Exact test p=0.012). Inclusion of a figure that resembled a CONSORT diagram was uncommon prior to 2000 and was found in only 5 (16.7%) trials, in comparison to 14 (56%) of trials published after 2000 (Fishers Exact test, p =0.0014).

To address the hypothesis that older trials had less effective compactor control groups, we tallied the use of placebo comparator versus use of another proven principal comparator in trials published before or after 2000. Among the control groups for trials published prior to 2000, 18 (60%) received placebo, 9 (30%) usual care or standard therapy and 3 (10%) an active comparator. After 2000, 16 (64%) of control groups received placebo, 7 (28%) usual care or standard therapy and 2 (8%) active comparator. Placebo versus active treatment comparator does not explain the increase trend in null results ($\chi 2$= .001, df=1, p=.979).

In recent years, NHLBI has become more active in managing clinical trials. Less than half of the trials published prior to 2000 (43%) were funded by a cooperative agreement or contract (n=13); the remaining studies were funded through an investigator-initiated grant. After 2000, 80% were funded through a cooperative agreement or a contract from NHLBI (n=20) whereas only 5 (20%) were funded by an investigator-initiated grant (χ^2= 6.19, df=1, p=0.013).

To investigate the effect of industry co-sponsorship we tabulated sponsorship for all reports. Unfortunately, industry co-sponsorship was not always reported prior to 2000 and journals did not uniformly require disclosure. After 2000, when the International Committee of

Medical Journal Editors (ICMJE) asked for disclosure, it became apparent that industry co-sponsorship is very common. In our sample, 23 of 25 (92%) of the NHLBI trials published after 2000 had partial industry sponsorship or contribution of medications. All but two of these trials obtained null results. We also looked at previous financial relationships between investigators and industry. Prior to 2000, these relationships were reported in only 1 of the 30 trials (3%). Even after 2000, 28% of the studies did not include a disclosure section. But among articles that included disclosures, there was a financial consulting relationship between at least one author and industry in all (100%) of the cases.

Table 3 breaks down the trials by benefit, harm, or null results on the primary outcome. Of the 30 trials that had not pre-registered (published prior to 2000), 17 reported a significant benefit for their primary outcome (57%), 1 showed harm and 12 were null (40%). Six trials did not report total mortality and 5 were not powered for total mortality. However, 5 showed that the treatment significantly reduced total mortality, 13 were null and 1 showed harm. For the 25 trials that had registered (published after 2000), 2 showed a benefit for the primary outcome (8%), 1 showed harm and 22 were null (88%). For total mortality, 22 were null, 2 showed harm, and 1 might have shown benefit had it been allowed to complete follow-up. There were significantly fewer trials with a significant benefit after 2000 than prior to 2000 ($\chi2$ =1, 12.2, p=0.0005).

Analysis of Primary Outcome. Figure 2 summarizes relative risks of the primary outcome for trials published prior to 2000 that had not registered prior to publication (left panel) and those published after 2000 that had registered (right Panel). Only 23 trials of the 30 trials published before 2000 included in the meta-analysis; 7 were excluded from the analysis because they only had continuous primary outcomes. A total 14 of 23 trials showed a significant benefit of intervention on the primary outcome. It should be noted that CPPT and CIS were coded as null in this analysis. In each of these two studies, the investigators applied a one tailed test, while their primary design papers called for two tailed tests. In both studies the effect size was null when re-calculated using a two-tailed test. Averaged across studies using meta-analysis, there was a significant benefit of treatment on the primary outcome (RR=0.81, 95% CI 0.73, 0.89). When

weighted by sample size, the RR was 0.77 (95% CI 0.71, 0.83). If analyzed separately for studies using cardiovascular events or angiographic exams as the primary outcome, the pooled effects were similar to the effects for all studies. However, when the primary outcome was not an event or angiographic outcome (such as blood pressure, medication assignment), the pooled effects were larger but null (RR = 0.77 95% CI 0.58, 1.08).

The right hand panel of Figure 2 summarizes the relative risks of trials published after year 2000 when all trials were registered. All the studies that were registered prior to publication analyzed primary outcomes that were a cardiovascular event or angiographic outcomes; none analyzed blood pressure or medication assignment alone. Two of the 25 pre 2000 trials were excluded because they had only continuous primary outcomes. Among the 23 trials, reporting binary primary outcomes, only 2 showed a significant benefit of treatment (PREVENT & SANDS). The pooled meta-analysis for primary outcomes was RR=0.97 (95% CI = 0.93, 1.01). When weighted by sample size, the pooled RR for primary outcomes was 0.98 (95% CI = 0.94, 1.02). Two studies reported borderline or significant harm for their primary outcome – AFFIRM and WHI for estrogen-progestin.

Mortality Outcomes. Figure 3 displays outcomes for total mortality, weighted by the standard error, for studies that had not registered prior to publication (left panel) and those that had registered (right panel). In the era prior to registration, 24 trials reported all cause-mortality and 5 reported significant reductions in total mortality (25%), 18 were null (71%) and one (CAST) reported significant harm. The pooled RR for total mortality was 0.92 (95% CI 0.84, 1.01) and when weighted by sample size the pooled RR was 0.92 (95% CI 0.86, 1.00).

Following the year 2000 when all trials had registered, no study showed a significant benefit for total mortality (pooled RR was 1.01 (0.98, 1.04) RR weighted by sample size = 1.01 (95% CI 0.97, 1.05)). However, there may have been a mortality benefit from the PREVENT study. The PREVENT was stopped early because of significant benefit achieved for the primary outcome. Although total mortality was null, this study could be counted as reducing total mortality had the original study follow-up period been completed. Two trials (ACCORD-

Diabetes & WAVE) suggested a significant harm for all-cause mortality and AFFIRM was borderline for harm. SANDS showed a significant protective benefit for carotid IMT and was null for mortality; however the significantly higher number of adverse events among participants assigned to treatment caused study authors to suggest that long-term outcomes might not be favorable. Overall, differences between the probability of reporting a significant benefit for all-cause mortality did not significantly decrease after registration was required ($\chi_{2=}$1.14, df=1, p=0.287), although no trial showed an all-cause mortality benefit in the era when trial registration was required.

Examination of Other Outcomes

Prospective declaration of the primary outcome variable is important because it eliminates the possibility of selecting for reporting an outcome among many different measures included in the study. In order to investigate this issue, we looked at the statistical significance of other variables not declared as the primary outcomes for preregistered studies. Among the 25 preregistered trials published in 2000 or later, 12 reported significant, positive effects for cardiovascular-related variables other than the primary outcome. Importantly, almost half of the trials might have been able to report a positive result if they had not declared a primary outcome in advance. Had the prospective declaration of a primary outcome not have been required, it is possible that the meta-analysis post-2000 would have looked very similar to the pre-2000 period.
