# Supplementary material for: Likelihood of Null Effects of Large NHLBI Clinical Trials Has Increased over Time
Source: PLoS One. 2015 Aug 5;10(8):e0132382. doi: 10.1371/journal.pone.0132382 (PMC4526697; doi:10.1371/journal.pone.0132382)
Supplement: S3 Text — (PDF) [file pone.0132382.s010.pdf]

Acronyms

Note: Reference numbers and Acronyms and reference numbers for Tables 1 and 2

1. ACAPS	Asymptomatic Carotid Artery Progression Study
2. AMIS	Aspiring Myocardial Infarction Study
3. BAATAF	Boston Area Anticoagulation Trial for Atrial Fibrillation
4. BHAT	Beta-Blocker Heart Attack Trial
5. Carotid	Aspirin plus dipryridamole to prevent restenosis after carotid endarterectomy
6. CASCADE study	Cardiac Arrest in Seattle: Conventional Versus Amiodarone Drug Evaluation
7. CAST	Cardiac Arrhythmia Suppression Trial
8. CDP	Coronary Drug Project
9. CIS	Coronary Intervention Study
10. CLAS	Cholesterol-lowering atherosclerosis study
11. CPPT	Coronary Primary Prevention Trial
12. FATS	Familial Atherosclerosis Treatment Study
13. FEN PHEN	(Fenfluramine & Phentemrine) Cardiovascular System in Obesity: Effect of Treatment
14. Fish Oil	Fish oil on blood pressure among mild hypertensive subjects
15. HCP	Hypertension Control Program
16. HDFP	Hypertension Detection and Follow-up Program
17. HPT	Hypertension Prevention Trial
18. KCL	Potassium chloride on blood pressure in hypertensive men
19. MILIS	Multicenter Investigation of Limitation of Infarct Size
20. MITIT	Myocardial Infarction Triage and Intervention Trial
21. MRFIT	Multiple Risk Factor Intervention Trial
22. Myocarditis	Immunosuppressive therapy for myocarditis
23. PEPI	Postmenopausal Estrogen/Progestin Interventions Study
24. PHYSCN_HLTH	Physician’s Health Study
25. SCRIP	Stanford Coronary Risk Intervention Project

26. SHEP	Systolic Hypertension in the Elderly Program
27. SOLVD	Studies of Left Ventricular Dysfunction
28. Thrombo	Late thrombolytic therapy preserves left ventricular function
29. TIMI	Thrombolysis in myocardial infarction
30. TOMHS	Treatment of Mild Hypertension Study
31. ACCORD - BP	Action to Control Cardiovascular Risk in Diabetes – Blood Pressure
32. ACCORD - Diabetes	Action to Control Cardiovascular Risk in Diabetes – Diabetes
33. ACCORD - Lipid	Action to Control Cardiovascular Risk in Diabetes - Lipids
34. ACES	Azithromycin and Coronary Events Study
35. AFFIRM	Atrial Fibrillation Follow-up Investigation of Rhythm Management
36. AIM-HIGH	Atherothrombosis Intervention in Metabolic Syndrome with Low HDL/High Triglycerides Impact on Global Health
37. ALLHAT-BP	Antihypertensive and Lipid-Lowering Treatment-Amlodipine
38. ALLHAT-Dox	Antihypertensive and Lipid-Lowering Treatment-Doxazosin
39. ALLHAT-LLT	Antihypertensive and Lipid-Lowering Treatment-Pravastatin
40. Alpha Omega	Alpha Omega Trial: Study of Omega-3 Fatty Acids and Coronary Mortality
41. ENRICHD	Enhancing Recovery in Coronary Heart Disease Patients
42. ERA	Estrogen Replacement and Atherosclerosis
43. IMMEDIATE	Initial Myocardial Metabolic Enhancement During Initial Assessment and Treatment in Emergency care
44. MAGIC	Magnesium in Coronaries (MAGIC)
45. PEACE	Prevention of Events With Angiotensin-Converting Enzyme Inhibitor Therapy
46. PREVENT	Prevention of Recurrent Venous Thromboembolism
47. SANDS	Stop Atherosclerosis in Native Diabetics Study
48. SCD-HeFT	Sudden Cardiac Death in Heart Failure Trial
49. WACS	Women's Antioxidant Cardiovascular
50. WAVE	Women’s Angiographic Vitamin and Estrogen trial
51. WELL HART	Women’s Estrogen-Progestin Lipid-Lowering Hormone

	Atherosclerosis Regression Trial
52. WHI-E	Women's Health Initiative- Estrogen
53. WHI-EP	Women's Health Initiative- Estrogen-Progestin
54. WHS-ASA	Women's Health Study- Aspirin
55. WHS-E	Women's Health Study- Vitamin E
