# Supplementary material for: Likelihood of Null Effects of Large NHLBI Clinical Trials Has Increased over Time
Source: PLoS One. 2015 Aug 5;10(8):e0132382. doi: 10.1371/journal.pone.0132382 (PMC4526697; doi:10.1371/journal.pone.0132382)
Supplement: S4 Text — (PDF) [file pone.0132382.s011.pdf]

Listing of Studies Included in Meta Analysis

1. Furberg CD et al. Effect of lovastatin on early carotid atherosclerosis and cardiovascular events. Asymptomatic Carotid Artery Progression Study (ACAPS) Research Group. Circulation. 1994; 90:1679-1687. [ACAPS]

2. Aspiring Myocardial Infarction Study Research Group. A randomized, controlled trial of aspirin in persons recovered from myocardial infarction. JAMA. 1980; 243(7): 661-669. [AMIS]

3. The Boston Area Anticoagulation Trial for Atrial Fibrillation in patients with nonrheumatic atrial fibrillation. NEJM. 1990; 323(22): 1505-1511. [BAATAF]

4. Beta-Blocker Heart Attack Trial Research Group. A randomized trial of propanol in patients with acute myocardial infarction: Mortality Results. JAMA. 1982; 247(12): 1707-1714. [BHAT]

5. The CASCADE Investigators. Randomized antiarrhythmnic drug therapy in survivors of cardiac arrest (the CASCADE study). American Journal of Cardiology. 1993; 72:280-287. [CASCADE]

6. Harker LA, et al. Failure of aspirin plus dipryridamole to prevent restenosis after carotid endarterectomy. Annals of Internal Medicine. 1992; 116(9): 731-736. [Carotid]

7. Echt DS, et al. Mortality and morbidity in patients receiving encainide, flecainide, or placebo. The Cardiac Arrhythmia Suppression Trial. NEJM. 1991; 324(12): 781-788. [CAST]

8. The Coronary Drug Project Research Group. Clofibrate and niacin in coronary heart disease. JAMA. 1975; 231(4): 360-381. [CDP]

9. Canner PL et al. Fifteen year mortality in coronary drug project patients: Long-term benefit with niacin. JACC. 1986; 8(6): 1245-1555. [CDP – long-term follow-up]

10. Brensike JF et al. Effects of therapy with cholestyramine on progression of coronary arteriosclerosis: results of the NHLBI Type II Coronary Intervention Study. Circulation. 1984; 69:313-324. [CIS]

11. Blankenhorn DH et al. Beneficial effects of combined colestipol-niacin therapy on coronary artherosclerosis and coronary venous bypass grafts. JAMA. 1987; 257(23): 3233-3240. [CLAS]

12. Lipid Research Clinics Program. The Lipid Research Clinics Coronary Primary Prevention Trial Results. JAMA. 1984; 251(3): 351-363. [CPPT]

13. Brown G et al. Regression of coronary artery disease as a result of intensive lipid-lowering therapy in men with high levels of apolipoprotein B. NEJM. 1990; 323(19): 1289-1298.[FATS]

14. Weintraub M et al. Long-term weight control study 1 (weeks 0 to 34). Clin Pharmacol Ther. 1992; 51:586-594.[FEN PHEN]

15. Stamler R et al. Final report of a four-year randomized controlled trial – the hypertension control program. JAMA. 1987; 257(11): 1485-1491. [HCP]

16. Hypertension Detection and Follow-up Program Cooperative Group. Five-year findings of the hypertension detection and follow-up program. Reduction in mortality of persons with high blood pressure, including mild hypertension. JAMA. 1979; 242(23); 2562-2571.[HDFP]

17. Hypertension Prevention Trial Research Group. The Hypertension Prevention Trial: Three-year effects of dietary changes on blood pressure. Arch Intern Med. 1990; 150:153-162. [HPT]

18. Roberts R et al. Effect of propranolol on myocardial-infarct size in a randomized blinded multicenter trial. NEJM. 1984; 311: 218-225. [MILIS]

19. Weaver WD et al. Prehospital-initiated vs Hospital-initiated thrombolytic therapy. JAMA. 1993; 270(10): 1211-1216. [MITIT]

20. Multiple Risk Factor Intervention Trial Research Group. Multiple Risk Factor Intervention Trial: Risk factor changes and mortality results. JAMA. 1982; 248(12): 1465-1477. [MRFIT]

21. Mason JW et al. A clinical trial of immunosuppressive therapy for myocarditis. NEJM. 1995; 333(5): 269-275. [Myocarditis]

22. Rentrop KP et al. Late thrombolytic therapy preserves left ventricular function in patients with collateralized total coronary occlusion: Primary end point findings of the second Mount Sinai-new York university reperfusion trial. JACC. 1989; 14:58-64. [Thrombo]

23. Morris MC et al. The effect of fish oil on blood pressure in mild hypertensive subjects: a randomized cross-over trial. Am J Clin Nutr. 1993; 57:59-64.[Fish oil]

24. Steering committee of the Physicians' Health Study Research Group. Final report on the aspirin component of the ongoing physicians' health study. NEJM. 1989; 321(3): 129-135. [Physicians' Health Study]

25. Grimm RH et al. The influence of oral potassium chloride on blood pressure in hypertensive men on a low-sodium diet. NEJM. 1990; 322(9): 569-574. [KCL]

26. Haskell wL et al. Effects of intensive multiple risk factor reduction on coronary artherosclerosis nad clinical cardiac events in men and women with coronary artery disease. The Stanford Coronary Risk Intervention Project (SCRIP). Circulation. 1994; 89: 975-990. [SCRIP]

27. Curb JD et al. Effect of diuretic-based antihypertensive treatment on cardiovascular disease risk in older diabetic patients with isolated systolic hypertension. JAMA. 1996; 276(23): 1886-1892. [SHEP]

28. The SOLVD Investigators. Effect of enalapirl on survival in patients with reduced left ventricular ejection fractions and congestive heart failure. NEJM. 1991; 325(5): 293-302. [SOLVD]

29. Neaton JD et al. Treatment of mild hypertension study: Final results. JAMA. 1993; 270 (6): 713-724. [TOMHS]

30. Chesebro JH et al. Thrombolysis in myocardial infarction trial (TIMI) , Phase 1: A comparison between intravenous tissue plasminogen activator and intravenous streptokinase. Clinical findings through hospital discharge. Circulation. 1987; 76: 142-154. [TIMI]

31. The TIMI study group. The thrombolysis in myocardial infarction (TIMI) trial. NEJM. 1985; 312(14):932-936.[TIMI]

32. The Writing Group for the PEPI trial. Effects of estrogen or estrogen/progestin regimens on heart disease risk factors in postmenopausal women. JAMA. 1995; 273(3): 199-208. [PEPI]

33. The ACCORD study group. Effects of intensive blood-pressure control in type 2 diabetes mellitus. NEJM. 2010; 362(17): 1575-1585. [ACCORD-BP]

34. The Action to Control Cardiovascular Risk in Diabetes Study Group. Effects of intensive glucose lowering in type 2 diabetes. NEJM. 2008; 358(24): 2545-2559. [ACCORD-Diabetes]

35. The ACCORD Study Group. Effects of combination lipid-therapy in Type 2 diabetes mellitus. NEJM. 2010; 362(17): 563-1574. [ACCORD-Lipid]

36. Grayston JT et al. Azithromycin for the secondary prevention of coronary events. NEJM. 2005; 352(16): 1637-1645.[ACES]

37. The Atrial Fibrillation Follow-up Investigation of Rhythm Management (AFFIRM) investigators. A comparison of rate control and rhythm control in patients with atrial fibrillation. NEJM. 2002; 347(23): 1825-1833. [AFFIRM]

38. Boden et al. Niacin in patients with low HDL cholesterol levels receiving intensive statin therapy. NEJM. 2011. 365(24): 2255. [AIM-HIGH]

39. Furberg CD et al. Major outcomes in high-risk hypertensive patients randomized to angiotensin-converting enzyme inhibitor or calcium channel blocker vs diuretic: The antihypertensive and lipid-lowering treatment to prevent heart attack trial (ALLHAT). JAMA 2002. 288(23):2981.[ALLHAT-BP]

40. Barry DR. Major cardiovascular events in hypertensive patients randomized to doxazosin vs chlorthalidone: The antihypertensive and lipid-lowering treatment to prevent heart attack trial (ALLHAT). JAMA. 2000. 283(15): 1967.[ALLHAT-Dox]

41. Furberg CD et al. Major outcomes in moderately hypercholesterolemic, hypertensive patients randomized to pravastatin vs usual care: The antihypertensive and lipid-lowering treatment to prevent heart attack trial (ALLHAT-LLT). JAMA. 2002. 288(23): 2998. [ALLHAT-LLT]

42. Kromhout D et al. n-3 fatty acids and cardiovascular events after myocardial infarction. NEJM. 2010. 363(21): 2015.[Alpha Omega]

43. Czajkowski SM et al. Effects of Treating Depression and Low Perceived Social Support on Clinical Events after Myocardial Infarction: The Enhancing Recovery in Coronary Heart Disease Patients (ENRICHD) Randomized Trial. JAMA. 2003. 289(23): 3106. [ENRICHD]

44. Herrington DM et al. Effects of estrogen replacement on the progression of coronary artery atherosclerosis. NEJM; 2000;343(8): 522-529. [ERA]

45. Selker HP et al. Out-of-hospital administration of intravenous glucose-insulin-potassium in patients with suspected acute coronary syndromes: The IMMEDIATE randomized controlled trial. JAMA. 2012. 301(18):1925. [IMMEDIATE]

46. Antman E M et al. Early administration of intravenous magnesium to high-risk patients with acute myocardial infarction in the Magnesium in Coronaries (MAGIC) Trial: A randomised controlled trial. Lancet. 2002. 360(9341): 1189.[MAGIC]

47. Braunwald E et al. Angiotensin-converting-enzyme inhibition in stable coronary artery disease. NEJM. 2004. 351(20): 2058. [PEACE]

48. Ridker PM, et al. Long-term, low-intensity warfarin therapy for the prevention of recurrent venous thromboembolism. NEJM. 2003. 348(15): 1425. [PREVENT]

49. Howard BV, Roman MJ, Devereux RB, Fleg JL, Galloway JM, et al. Effect of lower targets for blood pressure and LDL cholesterol on atherosclerosis in diabetes: The SANDS randomized trial. JAMA. 2008. 299(14): 1678-1689. [SANDS]

50. Fleg JL, Mete M, Howard BV, Umans JG, et al. Effect of statins alone versus statins plus ezetimibe on carotid atherosclerosis in Type 2 diabetes. JACC. 2008; 52(25): 2198-2205. [SANDS]

51. Bardy GH et al. Amiodarone or an implantable cardioverter-defibrillator for congestive heart failure. NEJM. 2005. 352(3): 225. [SCD-HeFT]

52. Cook NR et al. A randomized factorial trial of vitamins C and E and beta carotene in the secondary prevention of cardiovascular events in women: Results from the women's antioxidant cardiovascular study. Arch Intern Med.2007. 167(15): 1610. [WACS]

53. Hodis HN et al. Hormone therapy and the progression of coronary artery atherosclerosis in postmenopausal women. NEJM; 2003; 349;535-545. [WELL HART]

54. Rossouw JE et al. Risks and benefits of estrogen plus progestin in healthy postmenopausal women: Principal results from the women's health initiative randomized controlled trial. JAMA. 2002. 288(3): 321 [WHI-EP]

55. Anderson GL et al. Effects of Conjugated Equine Estrogen in Postmenopausal Women with Hysterectomy: The Women's Health Initiative Randomized Controlled Trial. JAMA. 2004. 291 (14): 1701 [WHI-E]

56. Ridker PM et al. A randomized trial of low-dose aspirin in the primary prevention of cardiovascular disease in women. NEJM. 2005. 352(13): 1293. [WHS-ASA]

57. Lee IM et al. Vitamin E in the primary prevention of cardiovascular disease and cancer. The women's health study: A randomized controlled trial. JAMA. 2005. 294(2): 56 [WHS-E]

58. Waters DD, et al. Effects of hormone replacement therapy and antioxidant vitamin supplements on coronary atherosclerosis in postmenopausal women: A randomized controlled trial. JAMA. 2002. 288(19):2432-2440. [WAVE]
